# Deep learning-based low-dose CT for adaptive radiotherapy of abdominal and pelvic tumors

**DOI:** 10.3389/fonc.2022.968537

**Published:** 2022-08-18

**Authors:** Wei Gong, Yiming Yao, Jie Ni, Hua Jiang, Lecheng Jia, Weiqi Xiong, Wei Zhang, Shumeng He, Ziquan Wei, Juying Zhou

**Affiliations:** ^1^ Department of Radiation Oncology, First Affiliated Hospital of Soochow University, Suzhou, China; ^2^ Real Time Laboratory, Shenzhen United Imaging Research Institute of Innovative Medical Equipment, Shenzhen, China; ^3^ Radiotherapy Business Unit, Shanghai United Imaging Healthcare Co., Ltd., Shanghai, China; ^4^ IRT Laboratory, United Imaging Research Institute of Intelligent Imaging, Beijing, China

**Keywords:** low dose CT, FBCT, CNCycle-GAN, adaptive radiotherapy, abdominal and pelvic tumors

## Abstract

The shape and position of abdominal and pelvic organs change greatly during radiotherapy, so image-guided radiation therapy (IGRT) is urgently needed. The world’s first integrated CT-linac platform, equipped with fan beam CT (FBCT), can provide a diagnostic-quality FBCT for achieve adaptive radiotherapy (ART). However, CT scans will bring the risk of excessive scanning radiation dose. Reducing the tube current of the FBCT system can reduce the scanning dose, but it will lead to serious noise and artifacts in the reconstructed images. In this study, we proposed a deep learning method, Content-Noise Cycle-Consistent Generative Adversarial Network (CNCycle-GAN), to improve the image quality and CT value accuracy of low-dose FBCT images to meet the requirements of adaptive radiotherapy. We selected 76 patients with abdominal and pelvic tumors who received radiation therapy. The patients received one low-dose CT scan and one normal-dose CT scan in IGRT mode during different fractions of radiotherapy. The normal dose CT images (NDCT) and low dose CT images (LDCT) of 70 patients were used for network training, and the remaining 6 patients were used to validate the performance of the network. The quality of low-dose CT images after network restoration (RCT) were evaluated in three aspects: image quality, automatic delineation performance and dose calculation accuracy. Taking NDCT images as a reference, RCT images reduced MAE from 34.34 ± 5.91 to 20.25 ± 4.27, PSNR increased from 34.08 ± 1.49 to 37.23 ± 2.63, and SSIM increased from 0.92 ± 0.08 to 0.94 ± 0.07. The P value is less than 0.01 of the above performance indicators indicated that the difference were statistically significant. The Dice similarity coefficients (DCS) between the automatic delineation results of organs at risk such as bladder, femoral heads, and rectum on RCT and the results of manual delineation by doctors both reached 0.98. In terms of dose calculation accuracy, compared with the automatic planning based on LDCT, the difference in dose distribution between the automatic planning based on RCT and the automatic planning based on NDCT were smaller. Therefore, based on the integrated CT-linac platform, combined with deep learning technology, it provides clinical feasibility for the realization of low-dose FBCT adaptive radiotherapy for abdominal and pelvic tumors.

## Introduction

With the improvement of the local control rate and 5-year survival rate of abdominal and pelvic tumors, the survival time of patients has been prolonged, and the side effects caused by radical radiotherapy or concurrent radiotherapy and chemotherapy have gradually emerged, such as persistent hematological toxicity (1), difficult to controlled radiation enteritis (2), radiation cystitis, femoral head necrosis, etc. Although the tumor is effectively controlled, patients have to face the pain and psychological burden brought by the cruel side effects of treatment. This has also become the main appeal of many tumor patients during their follow-up visits, but clinicians are often powerless at this moment. During radiotherapy, the position and shape of tissues and organs change greatly due to differences in respiration, intestinal peristalsis or intestinal gas, and the bladder filling. As well as changes in the patient’s outer contour due to weight loss and muscle atrophy, the position of the tumor or organs can be shifted. Therefore, IGRT of abdominal and pelvic tumors is particularly necessary. Most of the current IMRT treatment strategies for abdominal and pelvic tumors are to scan and locate CT before the first treatment to make a treatment plan. In the subsequent fractional treatment, the patient is firstly set up, and then radiotherapy is performed according to the same treatment plan. In order to avoid the missed exposure of the target volume, the most commonly used method is based on the clinical target volume (CTV) expansion, also known as the planned target volume (PTV) (3, 4). However, a larger PTV will inevitably bring serious side effects to the surrounding normal tissues (5), and the simple CTV-PTV expansion seems to be unable to meet the needs of modern precision radiotherapy. Therefore, how to kill tumor cells to the greatest extent while protecting the surrounding normal tissues as much as possible is an eternal topic in radiotherapy.

Adaptive radiotherapy (ART) technology is an effective method to deal with the above problems (6–8). It requires online guided images to determine the patient’s anatomical changes during the treatment process, and then adaptively adjusts the radiotherapy plan to reduce damage to organs at risk. Therefore, the accuracy of the guiding images becomes the prerequisite for ART. Existing image-guided techniques include ultrasound imaging (US), magnetic resonance imaging (MR), cone beam CT (CBCT), etc. US has the characteristics of no radiation, rich image information, and high soft tissue resolution. They are often used for image-guided radiotherapy for prostate cancer and bladder cancer (9, 10). Due to the high operation requirements and few types of diseases, it has not been widely used in clinical practice. MR images have high soft-tissue contrast and clear contour edges between organs, which are great advantages for image guidance of head and neck and abdominal and pelvic tumors (11, 12). However, there is no correlation between the gray value and electron density information in ultrasound images and MR images, and neither can be directly used for dose calculation in radiotherapy planning. From the dual perspectives of scanning time and dose calculation, CT based IGRT is popularized, and kilo-voltage cone beam CT (KV-CBCT) is the most commonly used CT image-guided method (13). Due to X-ray scattering and noise in the imaging area of KV-CBCT, the artifacts are large and cannot provide clear soft tissue images.

This study is based on an integrated CT-linac platform, which is equipped with a diagnostic-quality 16-slice CT imager. However, routine FBCT scans will inevitably bring additional radiation doses, which will increase the risk of a patient’s second primary tumor to a certain extent (14, 15). Therefore, even for IGRT, the imaging process should follow the As Low As Reasonably Achievable (ALARA) radiation protection safety concept, which requires that the imaging dose should be reduced as much as possible while meeting the imaging needs (16).

The method of reducing the current of X-ray tube is commonly used to reduce the radiation dose in FBCT imaging (17). While it leads to a decrease in the number of photons received by the detector, and the projection data is polluted by noise. The reconstructed CT images will have a lot of noise and artifacts, which will affect the identification of anatomical structures. Obviously, the image quality of FBCT relying on the reduced dose cannot be further involved in the process of IGRT. The existing low-dose CT image processing methods can be divided into three categories: one is to process the projection data. The noise in the projection data is statistically modeled and then filtered to denoise (18, 19); the second is an iterative reconstruction method. The system imaging geometry, photon statistical characteristics, noise distribution, etc. are used as objective function constraints, which are transformed to the image domain and projection domain multiple times to iteratively optimize the objective function (20, 21); the third is to denoise the reconstructed CT images in the image domain. The first two methods need to obtain the original projection data, which is difficult to implement. The third method directly processes the reconstructed CT images, which is fast and easy to integrate. With the rapid development of artificial intelligence, deep learning techniques have been increasingly applied to medical image denoising (22–24). Among them, Geng et al. proposed a learning strategy of the content-noise complementary learning (CNCL) through two convolutional networks to dnoise and content in medical images respectively, and had good denoising performance on medical images of different modalities (25). From the perspective of data acquisition, the cycle-consistent generative adversarial network (CycleGAN) adopts a dual-generator network structure and a cycle-consistent loss function to achieve network parameter training without paired data (26). This method greatly reduces the difficulty of collecting medical image data and thus becomes a commonly used network structure in low-dose CT image processing (27–29). Due to the limitation of medical data acquisition, simulated low-dose CT data are often used for experiments in the above studies. Furthermore, there are no studies evaluating low-dose FBCT images for ART in abdominal and pelvic tumors.

In this study, we propose a content-noise cycle consistent generative adversarial network (CNCycle-GAN) to restore the quality of low-dose CT images. We employ a content noise generator based on the CycleGAN framework to effectively remove noise artifacts from low-dose CT images while preserving the edge structure of the tissue. Then, we evaluate the quality of the low-dose CT network restoration images (RCT) through the objective evaluation parameters of the images, automatic delineation performance and dose calculation accuracy to judge whether the restored images can be applied to the ART workflow.

## Materials and methods

### Image acquisition

In this study, we used a CT-Linac, uRT-linac 506c from United Imaging Medical Technology Co., Ltd. for data acquisition. The machine integrates a 16-slice helical CT to acquire diagnostic-grade FBCT for IGRT. We selected the data of 76 patients with abdominal and pelvic tumors who received radiotherapy in the department of radiation oncology, First Affiliated Hospital of Soochow University from January to April 2021 for the study, and all patients had signed the informed consent. The patient age ranged from 27 to 77 years, with a median age of 58 years. Each patient underwent two helical scans with normal dose and low dose in IGRT mode in the middle of radiotherapy, and the two scans were completed between different fractions of treatment. Under normal dose scanning conditions, the tube voltage was 120 kV and the tube current was 233 mA. The average dose length product (DLP) was 485.11mGy*cm. Under low-dose scanning conditions, the tube voltage was 120 kV and the tube current was 24 mA. The DLP was 47.15mGy*cm. According to AAPM Report No96, the tissue weight factor of abdult in abdominal and pelvic is 0.015 mSv/(mGy*cm) (30). Therefore, the effective dose of the abdominal and pelvic cavity was 7.28mSv under the condition of normal dose scanning, and the effective dose under the condition of low-dose scanning was 0.71mSv. The spatial resolution of the reconstructed image was 0.9765*0.9765 *mm* ^2^, the layer thickness was 3mm, and the image size was 512*512.

### Network structure

**Figure 1:**
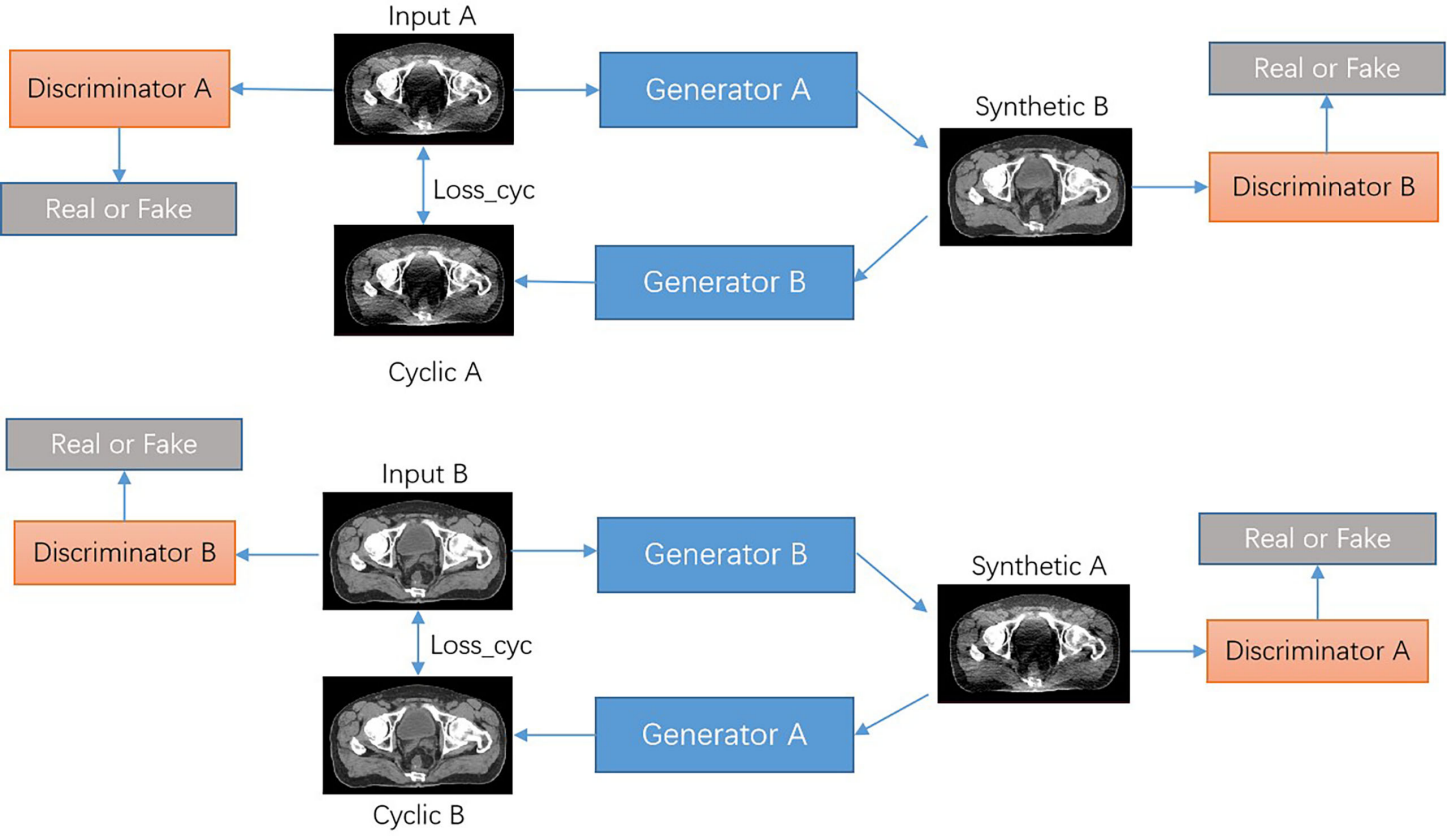
Architecture of the CNCycle-GAN network.

We proposed the CNCycle-GAN network to achieves bidirectional conversion between two domain images (A, low-dose CT images, LDCT; B, normal-dose CT images, NDCT). The network structure of CNCycle-GAN is shown in **Figure 1**. It uses two content noise convolutional networks as generators, *G*_*A*_:*A*→*B*, to realize the conversion of low-dose CT images to noraml-dose CT images; *G*_*B*_:*B*→*A*, to realize the conversion of normal-dose CT images to low-dose CT images. Each generator is adversarially trained *D*_*B*_ and *D*_*A*_ with the corresponding convolutional network discriminator.

**Figure 2:**
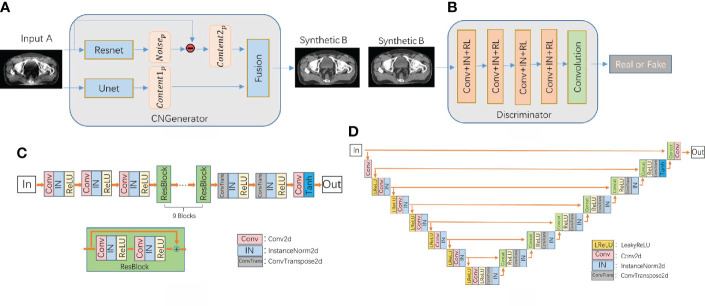
**(A)** CNCycle-GAN generator structure, **(B)** discriminator network structure diagram, **(C)** Resnet network structure in the generator, **(D)** Unet network structure in the generator.

As shown in **Figure 2**, the content noise generator is composed of a resnet predictor that predicts noise and a unet predictor that predicts content in parallel. The output images are reconstructed from the extracted features of the content predictor and noise predictor through a fusion network which composed of one convolutional layer. The resnet contains one convolution layer with a 7*7 kernel with stride 1; two down-sampling layers with a 3*3 kernel with stride 2 and channels 64 and 128; 9 residual blocks with a 3*3 kernel with stride 1; two up-sampling layers using ConvTranspose2d function with a 3*3 kernel with stride 2 and channels 256 and 128; one convolution layer with a 7*7 kernel with stride 1. The unet network has a 4*4 kernel stride 2 convolutional layer, 7 downsampling layers, 7 upsampling layers, and a deconvolution layer using the ConvTranspose2d function with 4*4 kernel stride 2. Among them, each downsampling layer contains a skip connection block, which is composed of the LeakReLU activation function, the convolutional layer InstanceNorm of 4*4 kernel stride 2, and the skip connections structure. The fusion network consists of a concatenation operation followed by a 1×1 convolution operation.

The discriminator uses PatchGAN in pix2pix (31) and output an array with 0 or 1 to determine whether the input image is fake or real.

Our CNCycle-GAN network uses Resnet and Unet in the generator to process the noise and content of the input image separately and then fuse them, which is superior to the 2D cyclegan network in both denoising effect and tissue edge details. In addition, due to the limitation of hardware resources during training, we did not adopt 3Dcyclegan as the recovery network.

For illustration, the generator *G*_*A* _ has the input LDCT images *I*_*A*_ and the output is the synthetic NDCT images *S*_*B*_=*G*_*A*_(*I*_*A*_). The input of the discriminator *D*_*B*_ is the synthetic NDCT images *S*_*B*_ and the real NDCT images *I*_*B*_. The two networks *G*_*A*_ and *D*_*B*_ play against each other during the training process, where *D*_*B*_ acts as a second classifier to determine whether the input images are a real LDCT images. On the other hand, the role of *D*_*A*_ is to increase the fidelity of the synthetic NDCT images, thereby fooling the discriminator. The above *G*_*A*_ and *D*_*B*_ training process is formulated as a min-max optimization task of the adversarial loss function *L*_*adv*_(*G*_*A*_,*D*_*B*_)


(1) $$\underset{G_{A}}{\min}\underset{D_{B}}{\max}L_{adv}(G_{A},D_{B})=\log(D_{B}(I_{B}))+\log(1-D_{B}(G_{A}(I_{A})))\;\;\;\;\;\;\;\;\;\;\;\;\;\;\;\;$$

Similarly, another set of generative adversarial losses *L*_*adv*_(*G*_*B*_,*D*_*A*_) can be formulated as:


(2) $$\underset{G_{B}}{\min}\underset{D_{A}}{\max}L_{adv}(G_{B},D_{A})=\log(D_{A}(I_{A}))+\log(1-D_{A}(G_{B}(I_{B})))\;\;\;\;\;\;\;\;\;\;\;\;\;\;\;\;\;$$

We used the cycle-consistent loss function *L*_*cyc*_(*G*_*A*_,*G*_*B*_) to ensure that a domain images can be restored as much as possible after the domain transformation by two generators. The loss function constrains the generation directions of the two generators while avoiding the direct interaction of the two domain images, enabling unsupervised network training.


(3) $$L_{cyc}\left(G_{A},G_{B}\right)={\left\|I_{A}-G_{B}\left(G_{A}\left(I_{A}\right)\right)\right\|}_{1}+{\left\|I_{B}-G_{A}\left(G_{B}\left(I_{B}\right)\right)\right\|}_{1}$$

It has been pointed out that increasing the identity loss can improve the stability of the generator (32), so we also introduced the identity loss:


(4) $$L_{iden}\left(G_{A},G_{B}\right)={\left\|I_{B}-G_{A}\left(I_{B}\right)\right\|}_{1}+{\left\|I_{A}-G_{B}\left(I_{A}\right)\right\|}_{1}$$

In summary, the total loss function of CNCycle-GAN is as follows:


(5) $$L(G_{A},G_{B},D_{A},D_{B})\;=L_{adv}(G_{A},D_{B})+L_{adv}(G_{B},D_{A})\;+\lambda_{cyc}L_{cyc}(G_{A},G_{B})+\lambda_{iden}L_{iden}(G_{A},G_{B})\;\;\;\;$$

where *λ*_*cyc*_ and *λ*_*iden*_ are the weights of *L*_*cyc*_(*G*_*A*_,*G*_*B*_) and *L*_*iden*_(*G*_*A*_,*G*_*B*_) respectively, which are used to control the importance of the corresponding loss.

### Experimental details

We randomly divided the NDCT images and LDCT images of 76 patients into a training group of 68 cases (90%) for network parameter training, and a test group of 8 cases (10%) for the evaluation of the image restoration performance of the network. Before network parameter training, the data needed to be preprocessed. We first normalized the pixel values of the CT images *I*_*ori*_ layer by layer to the range of (0, 1) according to the preset window width (WW) and window level (WL) to improve the model training speed. The normalized images *I*_*nor*_= (*I*_*ori*_+1000)/2800. In order to improve the robustness of the network and reduce the limitation of memory during training, we randomly cropped the normalized images into image blocks of 256*256 size, and performed data enhancement operations such as random rotation and random flip.

During the network training phase, we used ADAM optimization method to train all networks by minimizing the loss function (5), where *λ*_*cyc*_ was set to 20, *λ*_*iden*_ was set to 0.5, and epochs was set to 250. There were two stages to control the learning rate during training, we set the learning rate to 0.0002 for the first 150 epochs and linearly decreased it to zero in the following epochs. The mini-batch size was 4. The network parameters were initialized with values generated from a standard normal distribution before training. The proposed method was implemented using the PyTorch architecture, and NVIDIA Quadro RTX 4000 GPUs were used to train all networks. During the network testing stage, we used the sliding prediction strategy. The LDCT images of size 512*512 in the test group were sequentially overlapped and taken as the input of the generator *G*_*A*_,and the network output result *I*_*output*_ was obtained by weighted average of the output results of each block. Finally, inverse normalization operation was performed on the output of the network to obtain the RCT images, that is *I*_*RCT*_= *I*_output_*2800−1000 Image quality evaluation

In order to evaluate the image quality of RCT images, this paper adopted four objective evaluation indicators of mean absolute error (MAE) (33), mean square error (MSE), peak signal-to-noise ratio (PSNR) (34) and structural similarity (SSIM) (35) for quantitative analysis. The formulas are as follows:


(6) $$MAE_{X,Y}\;=\frac{1}{m}\sum_{i=1}^{m}\left|X_{i}-Y_{i}\right|\;\;\;\;\;\;\;\;\;\;\;\;\;\;\;\;\;\;\;\;\;\;\;\;\;\;\;\;\;\;\;\;\;\;\;\;\;\;\;\;\;\;\;\;$$

(7) $$MSE_{X,Y}\;=\frac{1}{m}\sum_{i=1}^{m}{\left|X_{i}-Y_{i}\right|}^{2}\;\;\;\;\;\;\;\;\;\;\;\;\;\;\;\;\;\;\;\;\;\;\;\;\;\;\;\;\;\;\;\;\;\;\;\;\;\;\;\;\;\;$$

(8) $$PSNR_{X,Y}\;=20\log_{10}\left(\frac{MAX}{\sqrt{MSE}}\right)\;\;\;\;\;\;\;\;\;\;\;\;\;\;\;\;\;\;\;\;\;\;\;\;\;\;\;\;\;\;\;\;\;\;\;\;$$

(9) $$SSIM_{X,Y}\;=\frac{\left(2\mu_{X}\mu_{Y}+C1)(2conv\left(X,Y\right)+C2\right)}{\left(\mu_{X}^{2}+\mu_{Y}^{2}+C1)(\sigma_{X}^{2}+\sigma_{Y}^{2}+C2\right)}\;\;\;\;\;$$

Among them,  *X*,*Y* are the two image to be compared, *m* is the total number of pixels in the image, *MAX* is the maximum value of the selected image, *μ* and *σ* are the mean and variance of the image, *conv* is the covariance of *X* and *Y*, *C*1 and *C*2 are used to maintain stability constant. Taking the NDCT images as the reference standard, the index parameters of the RCT images and the LDCT images were calculated respectively.

### Automatic delineation

Automatic delineation algorithms are often used in the ART process to improve the speed of delineation of organs at risk and target volumes. Therefore, the automatic delineation performance of RCT images is a key indicator of whether the images can be seamlessly embedded in the current ART workflow. We used the intelligent delineation system of UIH TPS for automatic delineation of the organ at risk and target volume. The system adopted a VBNet-based coarse segmentation and fine segmentation cascade network. The output result of the coarse segmentation network would guide the cropping of the original CT images, and the cropping result was input into the fine segmentation network to obtain the fine segmentation result. The loss function used by the cascaded network during training was the weighted average of the cross-entropy loss and the dice loss (36).

We used the intelligent delineation system of UIH TPS to automatically delineate the organ at risk and target volume in the RCT images of 6 cases of test group data, and then invited experienced clinicians to review and modify the delineation results on the RCT images. The automatic delineation results on RCT images were compared with the results of manual delineation by clinicians using the Dice Similarity Coefficient (DSC) and 95%Hausdorff distance to evaluate the automatic delineation performance of RCT images. Furthermore, in order to evaluate the performance difference of the automatic delineation function in TPS on the three sets of CT images, we performed the same delineation of the organ at risk and target volume on RCT, NDCT and LDCT, respectively. Taking the automatic contouring results of NDCT as reference, the automatic contouring results of LDCT and RCT were compared using DSC and 95%Hausdorff distance.

### Dose calculation

In the ART workflow, the dose calculation capability of CT-guided images determines whether the images can be used for ART. According to the delineation revised by the doctor and prescribed dose, we started the automatic planning function on the RCT images, and then copied the delineation and plan to the LDCT images and the NDCT images respectively to evaluate the clinical acceptability of each generated plan.

Taking the NDCT-based plan as a reference, the dose distribution and dose volume histogram (DVH) of the LDCT-based plan and the RCT-based plan were compared. For target dose, we assessed PTV for D90, D95 (dose to 90% and 95% of volume) and Dmean (mean dose), V95% and V100 (target volume to receive at least 95% and 100% of prescribed dose). For organ-at-risk doses, we assessed volumes receiving different dose levels, comparing V40 (V40 means the percentage of volume receiving 40Gy dose) and Dmax (maximum dose) for the rectum, bladder, and femoral head.

## Result

**Figure 3:**
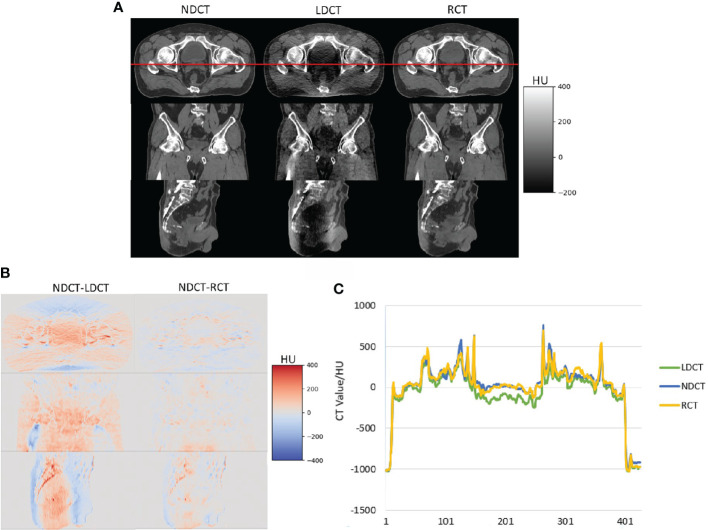
**(A)** Visual comparison results of NDCT, LDCT and RCT images, **(B)** image difference results of NDCT and LDCT and RCT respectively **(C)** the comparison results of CT values of the red profile lines which is located on the cross-sectional views of NDCT, LDCT and RCT.

**Figure 3** shows the image visual comparison results of NDCT, LDCT and RCT. The image difference results of NDCT and LDCT and RCT, and the comparison results of CT values of the red profile lines which is located on the cross-sectional views of NDCT, LDCT and RCT.

It can be seen from **Figure 3** that, from the visual comparison, compared with the LDCT images, the noise and artifacts on the RCT images are significantly reduced, and the tissue edge structures are well preserved. From the image CT value, the difference between RCT images and NDCT images is smaller. Therefore, the image quality of RCT images is closer to the NDCT images.

**Table 1** shows the objective evaluation indicators of each group of CT images. We performed statistical analysis on the data with two-sample equal variance t test. It can be seen from **Table 1** that compared with LDCT images, the MAE of RCT images is reduced from 34.34 ± 5.91 to 20.25 ± 4.27, the PSNR is increased from 34.08 ± 1.49 to 37.23 ± 2.63, and the SSIM is increased from 0.92 ± 0.08 to 0.94 ± 0.07. The P<0.01 of the above performance indicators indicates that the difference is statistically significant. Therefore, the image quality of RCT images is significantly improved from the objective evaluation index of images.

**Table 1:** Objective evaluation results of CT images quality.

	MAE(HU)	MSE(HU)	PSNR(dB)	SSIM
(LDCT,NDCT)	34.34 ± 5.91	3248.75 ± 1131.03	34.08 ± 1.49	0.92 ± 0.08
(RCT,NDCT)	20.25 ± 4.27	1815.48 ± 1300.81	37.23 ± 2.63	0.94 ± 0.07
P	<0.01	<0.01	<0.01	<0.01

The first row in **Table 2** shows the DSC results of the automatic delineation of the target volume and the organ at risk in the RCT images and the manual delineation of the clinician. Among them, the DSC of bladder, femoral head, and rectum reached more than 98%, indicating that the automatic delineation results of target area and organs at risk of RCT images are accurate enough to meet clinical needs.

**Table 2:** DSC comparison of CT image automatic delineation results.

	CTV	Bladder	Femoral Head R	Femoral Head L	Rectum
DSC(auto,manual)	0.96 ± 0.01	0.98 ± 0.02	0.98 ± 0.01	0.98 ± 0.01	0.98 ± 0.01
DSC(LDCT,NDCT)	0.79 ± 0.05	0.77 ± 0.14	0.95 ± 0.03	0.95 ± 0.03	0.18 ± 0.11
DSC(RCT,NDCT)	0.93 ± 0.02	0.95 ± 0.02	0.96 ± 0.04	0.97 ± 0.03	0.85 ± 0.05
P	<0.01	<0.01	0.77	0.71	<0.01

**Figure 4:**
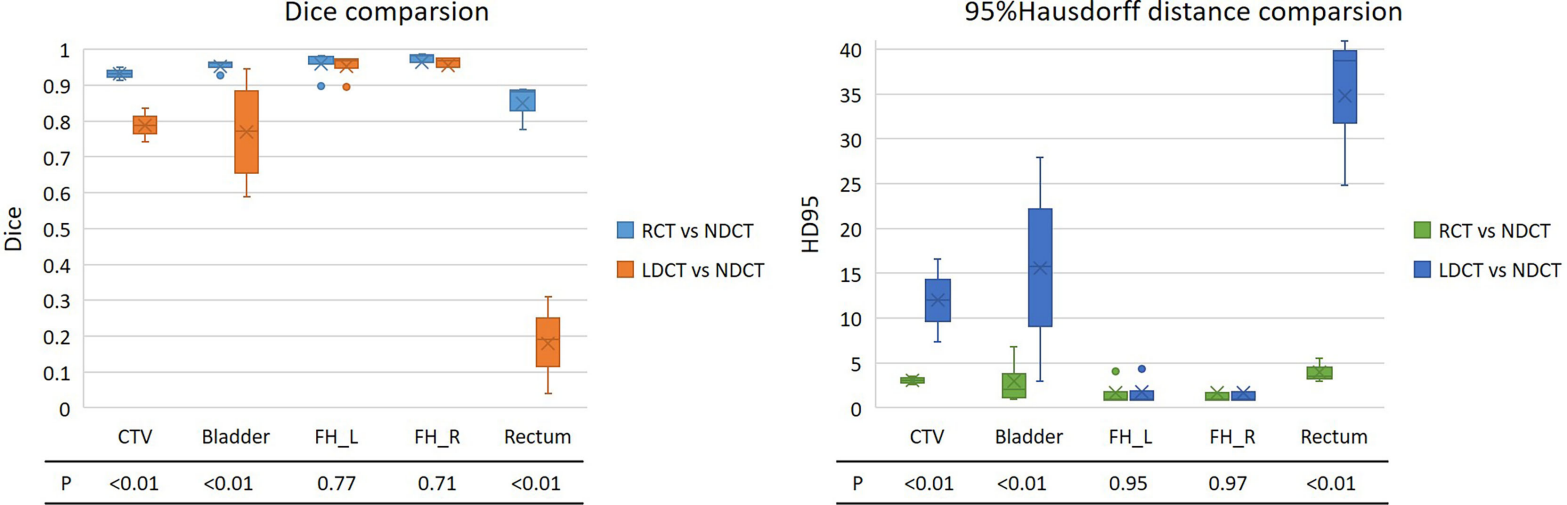
DSC and 95% Hausdorff distance results of the automatic delineation performance of RCT and LDCT with NDCT, respectively. The lower part of the figure is the statistical analysis results of the two-sample equal variance t test corresponding to the region of interest. FH_L and FH_R represent the left and right femoral heads.

Further to analysis, the second and third rows in **Table 2**, the two indicators of DSC and 95%Hausdorff in **Figure 4** show the difference between the automatic delineation results of RCT and LDCT and NDCT, respectively, and the corresponding two-sample equal variance t Test statistical analysis. The results show that for the automatic delineation of the femoral head, the DSC of RCT and LDCT are both above 0.95, and the HD95 are both less than 2. There is no significant difference in the automatic delineation results of the femoral head between RCT and LDCT. For automatic delineation of CTV and bladder, the DSC of RCT is above 0.93, and the HD95 is less than 3. Compared with LDCT, the automatic delineation results of RCT are closer to the automatic delineation results of NDCT. For the automatic delineation of the rectum, the DSC of LDCT is less than 0.20, and the HD95 is greater than 30. The automatic delineation performance of LDCT are poor, and there are cases where the automatic delineation algorithm cannot delineate normally at some LDCT slice images. The reason is that the noise and artifacts in the LDCT images of this layer have seriously affected the HU value of normal tissues, so that the automatic delineation algorithm cannot determine the boundary between normal organs and adjacent tissues.

For the automatic delineation of the femoral head, the P values for both DSC and HD95 are greater than 0.5, indicating that there is no significant difference between RCT and LDCT in the automatic delineation of the femoral head. While the P values of DSC and HD95 for CTV, bladder and rectum automatic delineation are all less than 0.01, indicating that there is a significant difference between the automatic delineation results of RCT and LDCT on CTV, bladder and rectum.

**Figure 5:**
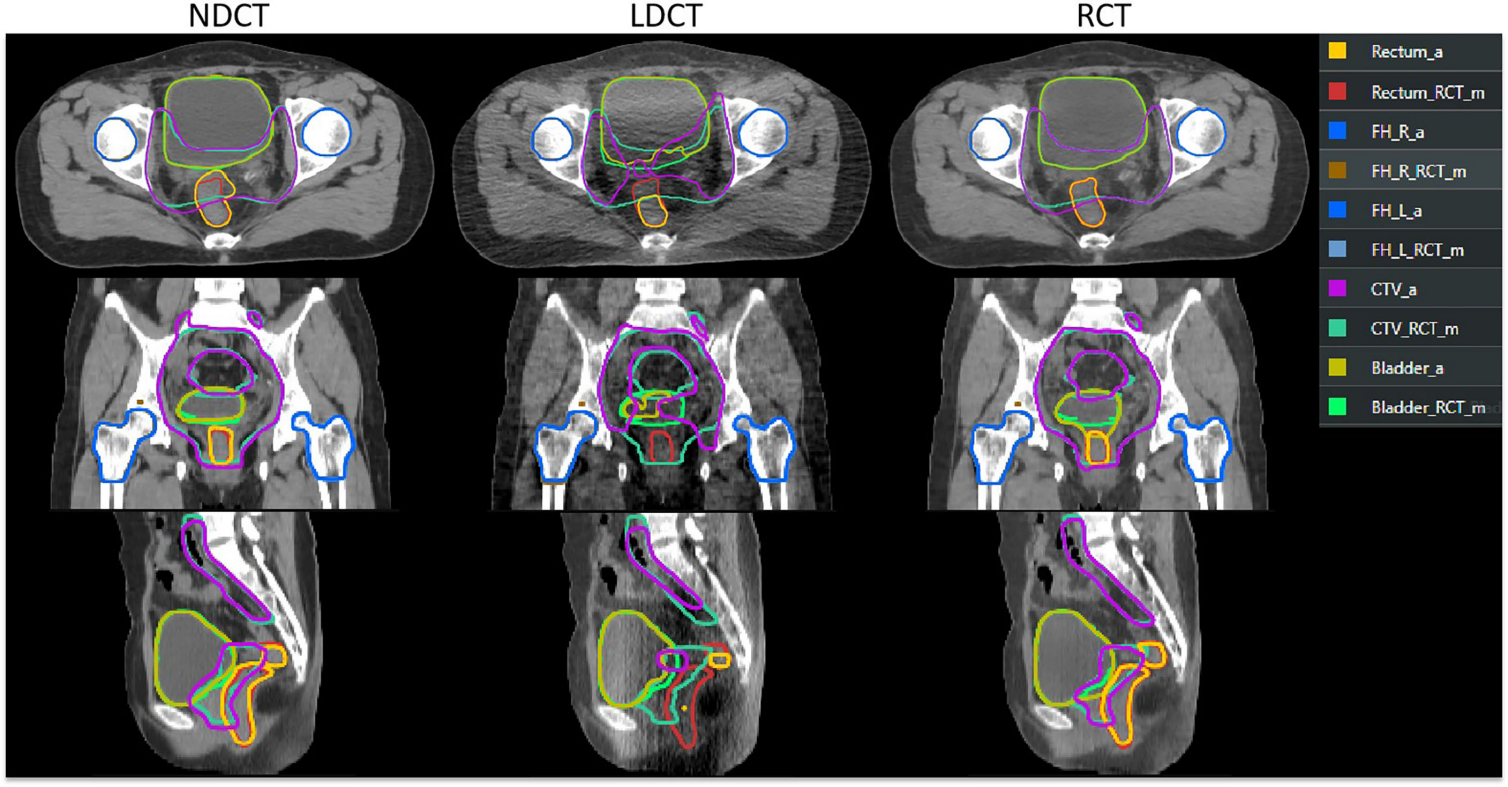
Comparison of automatic delineation results on different image sets with those manually modified by physicians on RCT. The results manually modified by the physician on the RCT were replicated on the LDCT and NDCT. ROI names suffixed with “_RCT_m” indicate that the physician manually modified the results on the RCT. ROIs with “_a” suffix indicate TPS automatic delineation results.

**Figure 5** shows the results of automatic delineation on different image sets compared to physician-modified delineation on RCT. Taking the doctor’s modified contour on RCT as a reference, it can be seen that the automatic contour results on the NDCT images and RCT images are similar to the doctor’s modified result; while the automatic contour results of the bladder and CTV on the LDCT images are quite different at the edge of the organ, so do the rectal contour. The automatic delineation of rectum in some patients are even wrong.

**Figure 6:**
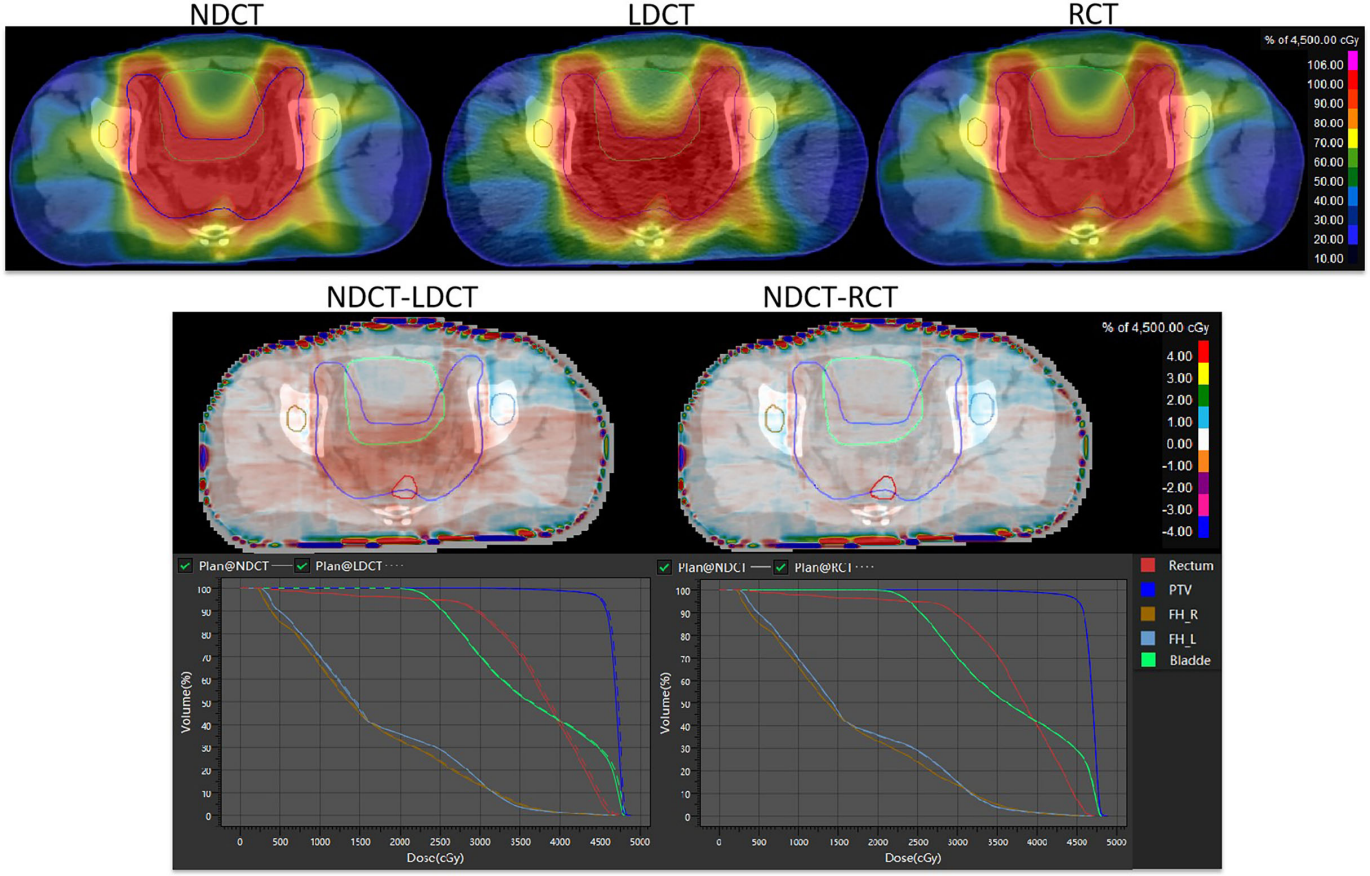
Comparison of dose distribution and dose-volume histogram results for NDCT-based plan, LDCT-based plan, and RCT-based plan.

The results of dose distribution and dose volume histograms between NDCT-based plan, LDCT-based plan and RCT-based plan are shown in **Figure 6**. It can be seen from the figure that the difference in dose distribution between the NDCT-based plan and the RCT-based plan is relatively small, and the DVH lines are almost overlap. The doses of NDCT-based plan and LDCT-based plan in rectum, bladder and PTV show small different. The results of dose distribution differences on axial slices in the second row show that in PTV, the dose difference between NDCT-based plan and LDCT-based plan reaches 1%, while the dose difference between NDCT-based plan and RCT-based plan is within 1%.

**Figure 7:**
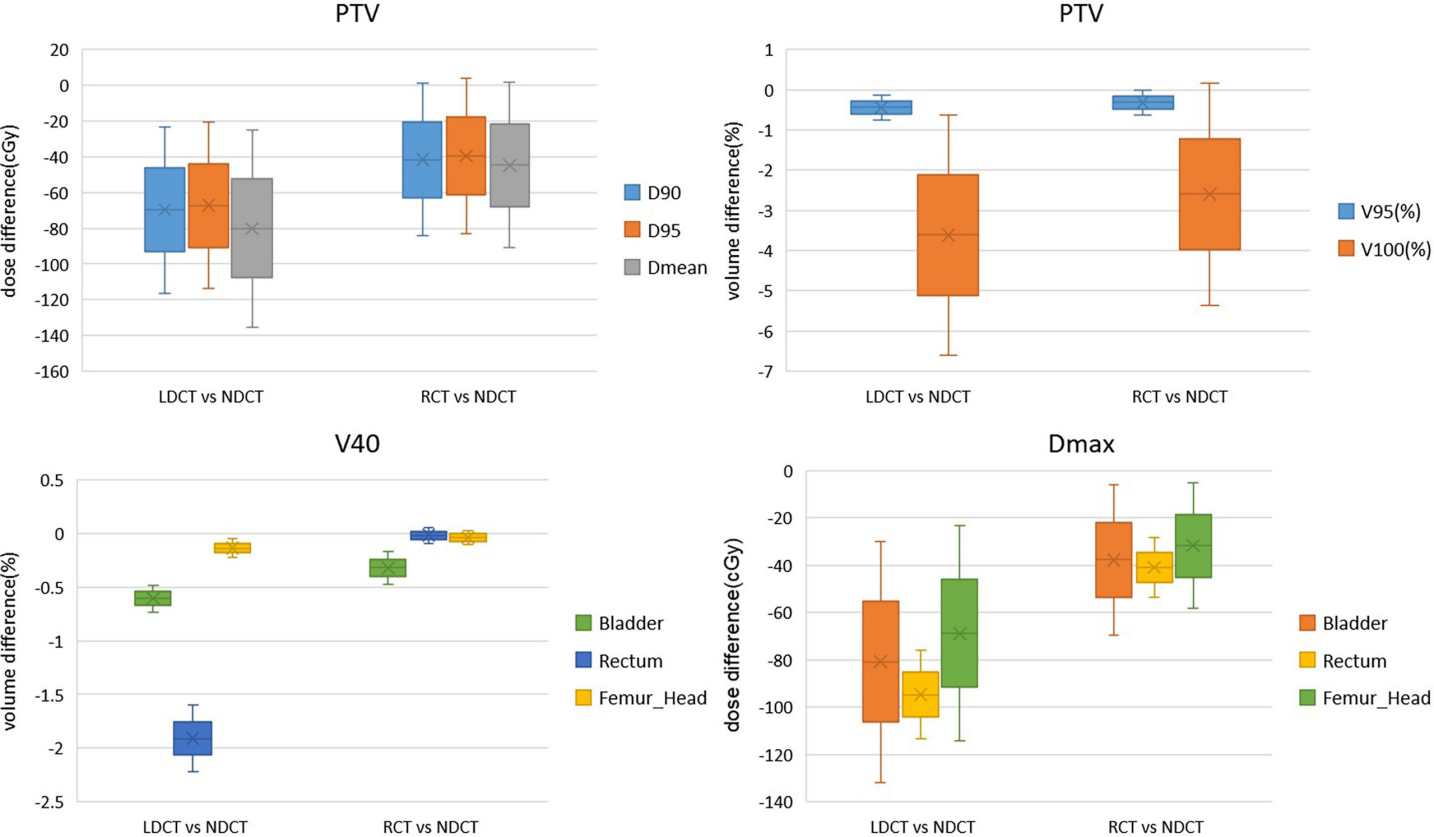
Dosimetric differences between RCT-based plan and NDCT-based plan and NDCT-based plan for PTV, bladder and femoral head plans.

The dosimetric differences of PTV, rectum, bladder and femoral head between RCT-based plan and LDCT-based plan and NDCT-based plan are shown in **Figure 7**. It can be seen from the figure that for the PTV dose statistics, the difference in D90, D95 and Dmean between the LDCT-based plan and the NDCT-based plan is as high as 65cGy, while the dose difference between the RCT-based plan and the NDCT-based plan is less than 45cGy. There was no significant difference between the LDCT-based plan and the RCT-based plan and the NDCT-based plan for the PTV volume that received 95% of the prescribed dose. For the PTV volume that received 100% of the prescribed dose, the difference between the RCT-based plan and the NDCT-based plan was 2.5% volume, which was smaller than the 3.6% volume difference between the LDCT-based plan and the NDCT-based plan. For the rectum, bladder and femoral head, it can be seen that the difference of V40 and Dmax between LDCT-based plan and NDCT-based plan is significantly larger than that of RCT-based plan and NDCT-based plan.

**Table 3**, **4** show that compared with the NDCT-based plan dose calculation results, the RCT-based plan and the LDCT-based plan have dose statistics P-values greater than 0.01 on PTV, bladder, rectum, and femoral head, indicating that both differences are not significant. The reason is that the CT values of RCT, LDCT and NDCT images are not very different, which can also be seen from the HU comparison results of the section line in **Figure 3**. Therefore, there was no significant difference in the dose calculation for RCT, LDCT and NDCT using the same plan.

**Table 3:** Two-sample equal variance t-test results of dose statistics for RCT-based plan and LDCT-based plan on PTV.

	D90	D95	Dmean	V95(%)	V100(%)
P	0.70	0.71	0.67	0.81	0.83

**Table 4:** Two-sample equal variance t-test results of dose statistics on bladder, rectum and femoral head for RCT-based plan and LDCT-based plan.

P	V95(%)	V100(%)
Bladder	0.28	0.55
Rectum	0.03	0.14
Femur Head	0.46	0.55

## Discussion

The linear accelerator uRT-linac 506c of United Imaging Medical Technology Co., Ltd. is the first radiotherapy equipment in the world that uses coaxial co-bed technology to integrate the accelerator and CT. Its diagnostic-grade FBCT can provide clear images for Monitoring of positioning errors during radiotherapy, tracking of tumor regression, etc. The abdominal and pelvic cavity is dominated by soft tissue structures, and traditional CBCT images cannot clearly display the boundaries of various tissues and organs. Therefore, the integrated FBCT imaging system brings convenience to image-guided radiotherapy for abdominal and pelvic tumors. New Zealand authors scanned daily CT (CT on-rails) in 5 pancreatic cancer patients treated with SBRT and found that, without violating the clinical limit of 5cc, the volume of stomach, duodenum, and small intestine received greater than 35Gy, was found to increase or remain constant during treatment. It demonstrating that diagnostic-grade CT imaging greatly contributes to the development of image-guided adaptive radiotherapy (37). However, guided radiotherapy based on diagnostic-grade CT is bound to bring the risk of excessive scanning radiation dose.

In recent years, with the wide application of low-dose CT scanning methods in the field of imaging diagnosis, the denoising methods of low-dose CT images based on deep learning have become a hot issue in this field (38, 39). In this study, we proposed a CNCycle-GAN network to solve the noise and artifacts in low-dose CT images of the abdominal and pelvis. **Figure 3** showed very intuitively that the noise and artifacts of the RCT images were significantly reduced. **Table 1** showed that compared with LDCT images, the MAE value of RCT images decreased by 41%, the PSNR value increased by 9.2%, and the SSIM value increased by 2.1%. Moreover, compared with the existing low-dose CT images restoration methods based on CycleGAN with the same multiple dose reduction (40), our proposed method had achieved the same images restoration performance a and the results on PSNR were even outperforms existing methods. The above results showed that the image quality of RCT images was closer to that of normal dose CT images, which laid a research foundation for subsequent image segmentation and planning.

Solving the problem of image quality is only the first step of adaptive radiotherapy. How to apply RCT images to image segmentation and planning is the more concerned issue of adaptive radiotherapy. As can be seen from **Figure 4** and **Figure 5**, the three groups of CT images had almost no difference in the results of automatic delineation of the femoral head. But for the automatic delineation of the bladder and rectum, it was difficult to judge the boundary of the bladder and rectum due to the influence of noise and artifacts in LDCT images. This shows that the use of LDCT images alone cannot meet the requirements of clinical image delineation of multiple normal organs. Once the delineation of soft tissue structures such as bladder and rectum is involved, CT images with close to normal doses must be used. Through the comparison of RCT images and NDCT images, we found that the two showed high consistency in the automatic delineation of organs at risk and target areas. Among them, the DSC of the bladder reached 95%. Even though the automatic delineation of the rectum required some minor modifications at some layers, it was sufficiently accurate compared to most model performance. The research on intelligent target delineation based on deep learning in pelvic tumors is also relatively mature. United Imaging’s TPS already has target delineation models for cervical cancer, rectal cancer and other tumors, and has published relevant literature (41, 42). As shown in **Table 2**, the automatic delineation results of CTV in 2 cases of cervical cancer surgery and 1 case of rectal cancer surgery achieved 96% of the DSC with the doctor’s manual modification of the delineation results. Therefore, for RCT images, the intelligent delineation model can be directly used in clinical work to delineate organs at risk and target areas, especially for patients with standardized target areas after surgery. This process can replace manual delineation and further improve the work efficiency of image-guided radiotherapy and adaptive radiotherapy without affecting the accuracy of the target volume.

It’s well known that there are two key technologies for adaptive radiotherapy. The first is precision and the second is speed. Our ultimate goal is to ensure that PTV covers online-GTV. These varied strategies include automatic planning as a key step in adaptive radiotherapy. In this study, we used the automatic planning function to evaluate the clinical acceptability of the generated plans and the time of automatic planning for each of the three sets of CT sets in the test data according to the organ at risk and target volume modified by the doctor and the given prescribed dose. Not only the PTV but normal tissue, there is little difference in dose distribution between RCT-based plan and NDCT-based plan. The accuracy of RCT images involved in dose calculation is confirmed, which is also consistent with the results of most RCT images involved in dose calculation (43). We observed 4 patients with large bladder and/or rectal displacement triggering off-line adaptive planning on FBCT scans. In TPS, the RCT images are automatically delineated first, and the attending physician reviews and appropriately modifies the target area, then completes the automatic plan, and calculates the total time required for the entire process of “automatic segmentation + manual modification + automatic planning”. Statistics show that it took 15s for automatic segmentation, 60s for manual modification, and 30s for automatic planning, which took nearly 2 minutes in total. The time taken is the shortest among the different adaptive radiotherapy methods currently available in the field of radiotherapy (44). We deserve further discussion for the accuracy and time advantages of image segmentation and dose calculation deserve, as well as how to use RCT image-guided individualized adaptive radiotherapy in clinical work. In addition, there is currently no scientific basis for the use of deep learning low-dose CT images for dose calculation. We need to explore the accuracy of low-dose CT directly involved in the calculation of radiotherapy planning. Therefore, the realization of online ART still needs further research.

## Conclusion

In this study, we used an integrated CT-linac system equipped with diagnostic-grade FBCT as the medium of image-guided radiotherapy, which avoided the deformation error of CBCT in image-guided radiotherapy. The system utilizes the advantages of low-dose CT in soft tissue imaging and radiation dose to provide high-quality, low-dose CT images, effectively reducing the random effect caused by ionizing radiation, and reducing the probability of secondary tumors. The possibility of frequent image-guided radiotherapy can be achieved. We used the CNCycle-GAN network method to solve the problem of noise and artifacts in low-dose CT images of 1/10 normal dose. The image quality, automatic delineation performance and dose calculation accuracy were used to evaluate whether the restored image RCT could be applied to the ART workflow. The results showed that the low-dose images RCT restored by the network had significantly reduced noise and artifacts, and the image quality was comparable to the NDCT images. The delineation of the target volume and the organ at risk was similar to that of the clinician’s manual delineation, and its dose calculation results were very close to the dose calculation results of NDCT images. The method proposed in this paper effectively restores the image quality of low-dose CT images of abdominal and pelvic regions, and provides a solution for IGRT of low-dose FBCT images in the ART process of abdominal and pelvic tumors. With the rapid development of artificial intelligence, it is expected that lower-dose CT scans can be used as a medium for image-guided radiotherapy to make greater contributions to individualized and precise radiotherapy.

## Data availability statement

The original contributions presented in the study are included in the article/Supplementary Material. Further inquiries can be directed to the corresponding authors.

## Author contributions

WG drafted the manuscript. YY and HJ completed data collection. ZW completes the network design. WX trains the network. LJ provides advice on model training. JN for image quality assessment. WG completes the manual modification of the delineation. SH has done a plan optimization. WZ supervises and manages the project. ZW and JZ designed the study and provided guidance. All authors contributed to the article and approved the submitted version.

## Conflict of interest

Author WX is employed by Shanghai United Imaging Healthcare Co., Ltd. Author WZ is employed by Shanghai United Imaging Healthcare Co., Ltd.

The remaining authors declare that the research was conducted in the absence of any commercial or financial relationships that could be construed as a potential conflict of interest.

## Publisher’s note

All claims expressed in this article are solely those of the authors and do not necessarily represent those of their affiliated organizations, or those of the publisher, the editors and the reviewers. Any product that may be evaluated in this article, or claim that may be made by its manufacturer, is not guaranteed or endorsed by the publisher.

## References

[1] Iorio GC Iorio GC Spieler BO Spieler BO Ricardi U Ricardi U. The impact of pelvic nodal radiotherapy on hematologic toxicity: A systematic review with focus on leukopenia, lymphopenia and future perspectives in prostate cancer treatment. Crit Rev Oncol Hematol (2021) 168:103497. doi: 10.1016/j.critrevonc.2021.103497 34666186

[2] Fernandes D Andreyev H. Gastrointestinal toxicity of pelvic radiotherapy: Are we letting women down? Clin Oncol (2021) 33(9):591–601. doi: 10.1016/j.clon.2021.04.009 33985867

[3] Bunt L Jürgenliemk-Schulz IM Kort G Roesink JM Tersteeg R Heide U. Motion and deformation of the target volumes during IMRT for cervical cancer: What margins do we need? Radiother Oncol (2008) 88:233–40. doi: 10.1016/j.radonc.2007.12.017 18237798

[4] Meijer GJ Rasch C Remeijer P Lebesque Joos V. Three-dimensional analysis of delineation errors, setup errors, and organ motion during radiotherapy of bladder cancer. Int J Radiat Oncol Biol Phys (2003) 55:1277–87. doi: 10.1016/S0360-3016(02)04162-7 12654438

[5] Schmid MP Mansmann B Federico M Dimopoulous J Ptter R. Residual tumour volumes and grey zones after external beam radiotherapy (with or without chemotherapy) in cervical cancer patients. Strahlenther Onkol (2013) 189(3):238–44. doi: 10.1007/s00066-012-0260-7 23344563

[6] Jong RD Crama KF Visser J Wieringen NV Wiersma J Geijsen ED. Online adaptive radiotherapy compared to plan selection for rectal cancer: quantifying the benefit. Radiat Oncol (2020) 15:1–9. doi: 10.1186/s13014-020-01597-1 PMC7371470 32641080

[7] Yan D Vicini F Wong J Martinez A. Adaptive radiation therapy. Phys Med Biol (1997) 42(1):123–32. doi: 10.1088/0031-9155/42/1/008 9015813

[8] Yan D. Adaptive radiotherapy: merging principle into clinical practice. Semin Radiat Oncol (2010) 20:79–83. doi: 10.1016/j.semradonc.2009.11.001 20219545

[9] Li M Ballhausen H Hegemann N-S Ganswindt U Manapov F. A comparative assessment of prostate positioning guided by three-dimensional ultrasound and cone beam CT. Radiat Oncol (2015) 10:82. doi: 10.1186/s13014-015-0380-1 25890013 PMC4465303

[10] Li M Ballhausen H Hegemann NS Reiner M Tritschler S Gratzke C. Comparison of prostate positioning guided by three-dimensional transperineal ultrasound and cone beam CT. Strahlenther Onkol (2017) 193:221–8. doi: 10.1007/s00066-016-1084-7 27928626

[11] Jsw A Sar B Mfb A. MRI-Guided adaptive radiotherapy for liver tumours: visualising the future. Lancet Oncol (2020) 21(2):e74-e82. doi: 10.1016/S1470-2045(20)30034-6 32007208

[12] Bayouth JE Low DA Zaidi H. MRI-Linac systems will replace conventional IGRT systems within 15years. Med Phys (2019) 46(9):3753–56. doi: 10.1002/mp.13657 31199516

[13] Boda-Heggemann J Lohr F Wenz F Flentje M Guckenberger M. kV cone-beam CT-based IGRT. Strahlenther Onkol (2011) 187:284–91. doi: 10.1007/s00066-011-2236-4 21533757

[14] Siiskonen T Kaijaluoto S Florea T. Imaging practices and radiation doses from imaging in radiotherapy. Phys Med (2017) 42:247–52. doi: 10.1016/j.ejmp.2017.03.012 28351527

[15] Ibbott GS. Patient doses from image-guided radiation therapy. Phys Med (2020) 72:30–1. doi: 10.1016/j.ejmp.2020.03.005 32197219

[16] Ding GX Alaei P Curran B Flynn R Gossman M Mackie TR. Image guidance doses delivered during radiotherapy: Quantification, management, and reduction: Report of the AAPM therapy physics committee task group 180. Med Phys (2018) 45(5):e84–e99. doi: 10.1002/mp.12824 29468678

[17] Bushberg JT Foster KR Hatfield JB Thansandote A Tell RA. IEEE Committee on man and radiation–COMAR technical information statement radiofrequency safety and utility smart meters. Health Phys (2015) 108:388–91. doi: 10.1097/HP.0000000000000217 25627954

[18] Jing W Lu H Liang Z Eremina D Manzione J. Noise properties of low-dose x-ray CT sinogram data in radon space. Proc SPIE - Int Soc Opt Eng (2008) 53(12):3327–41. doi:10.1088/0031-9155/53/12/018 10.1088/0031-9155/53/12/018 PMC2579780 18523346

[19] Manduca A Yu L Trzasko JD Khaylova N Kofler JM Mccollough CM. Projection space denoising with bilateral filtering and CT noise modeling for dose reduction in CT. Med Phys (2009) 36:4911–9. doi: 10.1118/1.3232004 PMC4108640 19994500

[20] Pickhardt PJ Lubner MG Kim DH Tang J Ruma JA Muñoz A. Abdominal CT with model-based iterative reconstruction (MBIR): initial results of a prospective trial comparing ultralow-dose with standard-dose imaging. Ajr Am J Roentgenol (2012) 199:1266. doi: 10.2214/AJR.12.9382 23169718 PMC3689212

[21] Fletcher Joel G Grant Katharine LR Fidler Jeff L Maria S Lifeng Y Jia W. Validation of dual-source single-tube reconstruction as a method to obtain half-dose images to evaluate radiation dose and noise reduction: phantom and human assessment using CT colonography and sinogram-affirmed iterative reconstruction (SAFIRE). J Comput Assist Tomogr (2012) 36:560. doi: 10.1097/RCT.0b013e318263cc1b 22992607

[22] Yang Q Yan P Zhang Y Yu H Shi Y Mou X. Low-dose CT image denoising using a generative adversarial network with wasserstein distance and perceptual loss. IEEE Trans Med Imaging (2018) 37(6):1348–57. doi: 10.1109/TMI.2018.2827462 PMC6021013 29870364

[23] Jiao F Gui Z Liu Y Yao L Zhang P. Low-dose CT image denoising via frequency division and encoder-dual decoder GAN. Signal Image Video Process (2021) 15:1907–15. doi: 10.1007/s11760-021-01935-0

[24] Naidich DP Marshall CH Gribbin C Arams RS McCauley DI. Low-dose CT of the lungs: preliminary observations. Radiology (1990) 175:729–31. doi: 10.1148/radiology.175.3.2343122 2343122

[25] Geng M Meng X Yu J Zhu L Jin L Jiang Z. Content-noise complementary learning for medical image denoising. IEEE Trans Med Imaging (2022) 41:407–19. doi: 10.1109/TMI.2021.3113365 34529565

[26] Zhu JY Park T Isola P Efros AA. Unpaired image-to-Image translation using cycle-consistent adversarial networks. IEEE International Conference on Computer Vision (ICCV) (2017), 2242–51. doi: 10.1109/ICCV.2017.244

[27] Li Z Huang J Yu L Chi Y Jin M. (2019). Low-dose CT image denoising using cycle-consistent adversarial networks, in: 2019 IEEE Nuclear Science Symposium and Medical Imaging Conference (NSS/MIC), 1–3. doi: 10.1109/NSS/MIC42101.2019.9059965

[28] Jg A Tsy B Jcy A Dong H. CycleGAN denoising of extreme low-dose cardiac CT using wavelet-assisted noise disentanglement. Med Image Anal (2021) 74:102209. doi: 10.1016/j.media.2021.102209 34450466

[29] Li Z Zhou S Huang J Yu L Jin M. Investigation of low-dose CT image denoising using unpaired deep learning methods. IEEE Trans Radiat Plasma Med Sci (2020) 5(2):224–34. doi: 10.1109/trpms.2020.3007583 PMC7978404 33748562

[30] McCollough CH Cody DD Edyvean S Geise RA Gould B Keat N. The measurement, reporting, and management of radiation dose in CT. AAPM Report (2008) 96:1–34. doi: 10.37206/97

[31] Isola P Zhu JY Zhou T Efros AA. Image-to-Image translation with conditional adversarial networks.IEEE Conference on Computer Vision and Pattern Recongnition (CVPR), (2017), 5967–67. doi: 10.1109/CVPR.2017.632

[32] Chai T Draxler RR. Root mean square error (RMSE) or mean absolute error (MAE). Geosci Model Dev Discuss (2014) 7:1525–34. doi: 10.5194/gmdd-7-1525-2014

[33] Huynh-Thu Q Ghanbari M. Scope of validity of PSNR in image/video quality assessment. Electron Lett (2008) 44:800–1. doi: 10.1049/el:20080522

[34] Zhou W Bovik AC Sheikh HR Simoncelli EP. Image quality assessment: from error visibility to structural similarity. IEEE Trans Image Process (2004) 13:600–12. doi: 10.1109/TIP.2003.819861 15376593

[35] Mu G Lin Z Han M Yao G Gao Y. (2019). Segmentation of kidney tumor by multi-resolution VB-nets, in: 2019 Kidney Tumor Segmentation Challenge, KiTS19. doi: 10.24926/548719.003

[36] Papalazarou C Klop GJ Milder MT Marijnissen JP Gupta V Heijmen B. CyberKnife with integrated CT-on-rails: System description and first clinical application for pancreas SBRT. Med Phys (2017). doi: 10.1002/mp.12432 28657157

[37] Reduced lung-cancer mortality with low-dose computed tomographic screening. N Engl J Med (2011) 365:395–409. doi: 10.1056/NEJMoa1102873 21714641 PMC4356534

[38] Nicolan B Greffier J Dabli D Forges HD Frandon J. Diagnostic performance of ultra-low dose versus standard dose CT for non-traumatic abdominal emergencies. Diagn Interv Imaging (2021) 102(6):379–87. doi: 10.1016/j.diii.2021.02.006 33714689

[39] Gu J Ye JC. AdaIN-switchable CycleGAN for efficient unsupervised low-dose CT denoising. arXiv (2020) 1:1–12. doi: 10.1109/TCI.2021.3050266

[40] Guo H Wang J Xia X Zhong Y Hu W. The dosimetric impact of deep learning-based auto-segmentation of organs at risk on nasopharyngeal and rectal cancer. Radiat Oncol (2021) 16(1):113. doi: 10.1186/s13014-021-01837-y 34162410 PMC8220801

[41] Ma CY Zhou JY Xu XT Guo J Han MF Gao YZ. Deep learning-based auto-segmentation of clinical target volumes for radiotherapy treatment of cervical cancer. J Appl Clin Med Phys (2021) 23(2):e13470. doi: 10.1002/acm2.13470 34807501 PMC8833283

[42] Zhao J Chen Z Wang J Xia F Zhang Z. MV CBCT-based synthetic CT generation using a deep learning method for rectal cancer adaptive radiotherapy. Front Oncol (2021) 11. doi: 10.3389/fonc.2021.655325 PMC8201514 34136391

[43] Lim-Reinders S Keller BM Al-Ward S Sahgal A Kim A. Online adaptive radiation therapy. Int J Radiat Oncol Biol Phys (2017) 99:994. doi: 10.1016/j.ijrobp.2017.04.023 28916139

[44] Kang E Koo HJ Yang DH Seo JB Ye JC. Cycle consistent adversarial denoising network for multiphase coronary CT angiography. Med Phys (2019) 46(2):550–62. doi: 10.1002/mp.13284 30449055

